# The RecA-Dependent SOS Response Is Active and Required for Processing of DNA Damage during *Bacillus subtilis* Sporulation

**DOI:** 10.1371/journal.pone.0150348

**Published:** 2016-03-01

**Authors:** Fernando H. Ramírez-Guadiana, Rocío del Carmen Barajas-Ornelas, Saúl U. Corona-Bautista, Peter Setlow, Mario Pedraza-Reyes

**Affiliations:** 1 Department of Biology, Division of Natural and Exact Sciences, University of Guanajuato, Guanajuato, Gto. 36050, México; 2 Department of Molecular Biology and Biophysics, UConn Health, 06030–3305, Farmington, Connecticut, United States of America; Institut de Génétique et Développement de Rennes, FRANCE

## Abstract

The expression of and role played by RecA in protecting sporulating cells of *Bacillus subtilis* from DNA damage has been determined. Results showed that the DNA-alkylating agent Mitomycin-C (M-C) activated expression of a P_*recA*_*-gfpmut3a* fusion in both sporulating cells’ mother cell and forespore compartments. The expression levels of a *recA-lacZ* fusion were significantly lower in sporulating than in growing cells. However, M-C induced levels of ß-galactosidase from a *recA-lacZ* fusion ~6- and 3-fold in the mother cell and forespore compartments of *B*. *subtilis* sporangia, respectively. Disruption of *recA* slowed sporulation and sensitized sporulating cells to M-C and UV-C radiation, and the M-C and UV-C sensitivity of sporangia lacking the transcriptional repair-coupling factor Mfd was significantly increased by loss of RecA. We postulate that when DNA damage is encountered during sporulation, RecA activates the SOS response thus providing sporangia with the repair machinery to process DNA lesions that may compromise the spatio-temporal expression of genes that are essential for efficient spore formation.

## Introduction

Sporulation in *Bacillus subtilis* is a developmental process that is triggered by nutrient depletion and high cell density, and serves as a strategy for population survival. An early morphological event in sporulation is the asymmetric cell division that gives rise to a two-compartment sporangium [1]. Distinct programs of gene expression are then directed in these two cells by compartment-specific RNA polymerase sigma (σ) factors [2]. The spores produced in this process exhibit minimal if any metabolism, but are highly resistant to heat, radiation, desiccation and chemical agents [3, 4]. Consequently, these spores can survive for very long periods [5, 6], until they encounter an appropriate environment allowing spore germination and resumption of cell growth [7].

Spontaneous or induced chromosomal damage in either compartment of the sporangium may compromise the successful accomplishment of the sporulation program in *B*. *subtilis*. Consequently, sporulating cells deploy a variety of mechanisms to sense and process DNA lesions that could interfere with proper spore formation [8–11]. These mechanisms include: 1) the DNA-damage scanning protein (DisA) which operates early in sporulation delaying this developmental program until genomic lesions have been eliminated; and 2) the nucleotide excision repair (NER) pathway that processes DNA-distorting or -crosslinking lesions produced by ultraviolet irradiation and mitomycin-C (M-C), respectively. Interestingly, the efficiency of the NER system is enhanced by the transcription-coupling repair (TCR) factor, Mfd, during *B*. *subtilis* sporulation [11]. Moreover, the UV-endonuclease YwjD, which possibly works in concert with the error-prone polymerases YqjH and/or YqjW also operates during sporulation to eliminate UV-promoted DNA damage [9, 10]. Of note, genes encoding UvrA and UvrB repair proteins as well as YqjW are part of the SOS regulon, a gene circuitry that is under transcriptional control by RecA and DinR [12, 13]. In exponentially growing *B*. *subtilis* cells, the presence of strand breaks promote the formation of RecN foci; moreover, viability is compromised in growing RecA-deficient cells [14]. Interestingly, sporangia lacking RecA exhibit aberrant morphologies, which likely affect nucleoid condensation and ultimately result in a low sporulation frequency [15]. Low levels of RecA have been detected in dormant spores; however, the RecA concentration increases during spore outgrowth and its contribution to DNA repair during this developmental stage has been demonstrated [16]. Thus, spores lacking RecA exhibit decreased resistance to DNA damaging factors such as heat, UV irradiation, hydrogen peroxide, ultrahigh vacuum desiccation and ionizing radiation [16–19]. A recent study revealed that YabT, a Hanks-type protein kinase, phosphorylates RecA during sporulation promoting the formation of RecA foci that can assemble into a nucleofilament in response to DNA damage. Interestingly, persistence of this protein structure beyond late sporulation results in abortive spore morphogenesis, suggesting that phosphorylated RecA may contribute to a checkpoint that delays or prevents the completion of spore development in sporangia with severely damaged chromosomes [20].

Although it has been shown that DNA replication is important for assembly of RecA in response to DNA damage and likely for SOS induction [21], there is no current information regarding the role of this protein when the progress of the transcription complex becomes impaired due to DNA damage. This is important since during endospore formation, *B*. *subtilis* does not replicate its chromosomes, although the transcriptional program that is essential for endospore development continues [2, 22, 23]. We report here that expression of *recA* is induced by DNA-damaging factors during sporulation, and in both sporangium compartments, and that the activation of the SOS response by RecA is required to eliminate DNA lesions inflicted by UV-C and M-C in *B*. *subtilis* sporangia.

## Materials and Methods

### Bacterial strains and growth conditions

The *B*. *subtilis* strains and plasmids used in this study are described in Table 1. Bacterial cultures were grown at 37°C in Luria-Bertani medium (LB, [24]), Penassay broth (PAB) (antibiotic medium 3; Difco Laboratories, Sparks, MD) or Difco sporulation medium (DSM, [25]) with shaking at 250 rpm. When appropriate, spectinomycin (100 μg/mL), neomycin (12.5 μg/mL) or erythromycin (5 μg/mL) was added to media.

**Table 1:** *B*. *subtilis* strains and plasmids used in this study.

Strain	Genotype and description^a^	Construction or source^b^
168	Wild type, *trpC2*	Laboratory stock
SL7360	Δ*recA*::*neo* (Neo)	Patrick Piggot
PERM938	Δ*mfd*::*tet* (Tet)	[11]
PERM1030	Δ*recA*::*neo* (Neo)	SL7360→168
PERM1033	Δ*mfd*::*tet*, Δ*recA*::*neo* (Tet Neo)	SL7360→PERM938
PERM1233	*spoVFA*::*lacZ* (Ery)	Laboratory stock
PERM1238	*amyE*::P_*recA*_-*gfpmut3a* (Spc)	pPERM1237→168
PERM1238a	*amyE*::*-gfpmut3a* (Spc)	pPERM1237a→168
PERM1280	Δ*recA*::*neo*, *spoVFA*::*lacZ* (Neo Ery)	SL7360→PERM1233
YB3001	*amyE*::*recA*-*lacZ* (Cm)	Ron Yasbin
LAS600	Δ*upp*	[31]
LAS523	+*dinR3* [*lexA*(Ind^-^)]	[31]
PERM1486	LAS600 *amyE*::*recA*-*lacZ* (Cm)	YB3001 → LAS600
PERM1487	LAS523 *amyE*::*recA*-*lacZ* (Cm)	YB3001 → LAS523
**Plasmid**		
pAD123	Shuttle *gfpmut3a* fusion vector (Amp Cm)	BGSC
pDR111	*amyE*::P_*hyper-spank*_ promoter (P_*hs*_) (Amp Spc)	David Rudner
pPERM1236	pAD123 containing the P_*recA*_*-gfpmut3a* construct (Amp Cm)	This study
pPERM1237	pDR111 containing the P_*recA*_-*gfp*mut3a construct (Amp Spc)	This study

^*a*^Selection markers are in parentheses. Antibiotics were used at the following concentrations: Neo, neomycin (12.5 μg/mL); Tet, tetracycline (10 μg/mL); Ery, erythromycin (5 μg/mL); Spc, spectinomycin (100 μg/mL); Amp, ampicillin (100 μg/mL) and Cm, chloramphenicol (5 μg/mL).

^*b*^ “X” → “Y” indicates that “strain Y” was transformed with DNA from “source X”.

BGSC, *Bacillus* Genetic Stock Center.

### Strains construction

Standard techniques were used for strain construction, including isolation of chromosomal and plasmid DNA as well as for bacterial transformation [26–28].

To generate a translational P_*recA*_-*gfpmut3a* gene fusion, a 492-bp EcoRI/BamHI fragment containing the *recA* promoter (-345 to +147 relative to the *recA* start codon) was PCR amplified using chromosomal DNA from *B*. *subtilis* 168. The oligonucleotide primers used for this reaction were 5’-GCGAATTCCAGGACCTGATGCTCAAG-3’ (forward) and 5’-GCGGATCCCAGTGCTGTATCAAGAGC-3' (reverse). Restriction sites (underlined) were included in the primers for cloning the amplified product between the EcoRI-BamHI sites of plasmid pAD123 to generate plasmid pPERM1236. This latter plasmid was cut with EcoRI and SphI and the resulting P_*recA*_-*gfpmut3a* DNA fragment was cloned between the same restriction sites of plasmid pDR111 (a generous gift from David Rudner), giving plasmid pPERM1237 (*amyE*::P_*recA*_-*gfpmut3a*). This plasmid was linearized and independently used to transform *B*. *subtilis* 168 to generate *B*. *subtilis* strains PERM1238 and PERM1238a, respectively.

Chromosomal DNA was isolated from *B*. *subtilis* YB3001 (*amyE*::*recA-lacZ*) and used to transform competent cells of *B*. *subtilis* strains LAS600 and LAS523, generating *B*. *subtilis* PERM1486 and PERM1487, respectively.

Chromosomal DNA from *B*. *subtilis* SL7360 (Δ*recA*::*neo*) was used to transform competent cells of *B*. *subtilis* strains168, PERM938 and PERM1233, generating strains PERM1030 (Δ*recA*), PERM1033 (Δ*mfd* Δ*recA*) and PERM1280 (Δ*recA*; *spoVFA*-*lacZ*), respectively. Proper single- and double-crossover events leading to the integration of vectors and/or to the inactivation of the appropriate genes were confirmed in all cases by PCR (data not shown).

### ß-galactosidase assays

ß-galactosidase activities during *B*. *subtilis* sporulation were determined as described by [29]. In brief, samples harvested from sporulating cultures were disrupted with lysozyme, centrifuged and the supernatant fluid was assayed for ß-galactosidase using ortho-nitrophenyl-ß-D-galactopyranoside (ONPG) as the substrate. This activity was assigned as the mother cell fraction, and consisted of enzyme from mother cells as well as lysozyme-sensitive forespores. The pellet fraction that contains lysozyme-resistant forespores present from 4 to 8 h after T_0_ (the time when the slopes of the logarithmic and stationary phases of growth intersected and defined as the time of initiation of sporulation) was subjected to spore coat removal [29], and a second lysozyme treatment was used to allow assay of the ß-galactosidase activity in forespores. All ß-galactosidase activities were expressed in Miller units [24], and the basal specific activities of ß-galactosidase in the RecA strain without a *lacZ* fusion during growth and sporulation were determined in parallel and subtracted from the data obtained for the strain carrying the *recA-lacZ* fusion. These corrections were always ≤ 10%.

### Microscopic analysis

*B*. *subtilis* cells were grown and induced to sporulate in DSM. 1 hour after T_0_, sporulating cell samples were supplemented with FM4-64 (Invitrogen) at a final concentration of 5μg/ml. Cell samples collected at the appropriate times were processed for microscopy essentially as described previously [11] and samples were observed under a ZEISS Axioscope A1 microscope equipped with an AxioCam ICc1. Image collection was carried out using AxioVision V 4.8.2 software and images were adjusted only for brightness and contrast. Excitation and emission wavelengths employed were 498 nm and 512 nm for GFP and 506 and 750 nm for FM4-64, respectively. More than 400 sporulating cells expressing the GFPmut3a protein (from ~600 cells analyzed) were counted in four different fields at 100☓ for each sporulation time point.

### Treatment of sporulating cells with DNA-damaging factors

*B*. *subtilis* strains were induce to sporulate in DSM at 37°C. At 4.5h after T_0_, the cultures were challenged with M-C or UV-C radiation as described previously [10]. Briefly, different doses of M-C were added to 2 mL cell samples and the cultures were incubated for additional 1 h before plating. Prior to UV-C treatment, cells were collected by centrifugation and washed with cold phosphate-buffered saline (PBS; 0.7% Na_2_HPO_4_, 0.3% KH_2_PO_4_, 0.4% NaCl [pH 7.5]). Cell samples (8 mL at an OD_600_ = 0.5) in PBS were stirred continuously and UV-C irradiated at room temperature. Artificial UV-C light (monochromatic 254 nm UV irradiation) was provided by a commercial low-pressure mercury arc lamp (model UVG-11; UV Products, Upland, CA). Cell survival after these treatments was determined by plating serial dilutions on solid LB medium, and colony-forming units were counted after 16 h of incubation at 37°C.

## Results and Discussion

### *recA* is induced by DNA damage during *B*. *subtilis* sporulation

Since RecA regulates the SOS response in *B*. *subtilis* [30], we generated a recombinant strain of *B*. *subtilis* to monitor *in situ* the transcriptional activation of the SOS response by DNA damage. To this end, the *gfp*mut3a reporter gene was fused to the *recA* promoter and the resulting P_*recA*_*-gfpmut3a* fragment was recombined into the *amyE* locus of *B*. *subtilis*.

**Fig 1:**
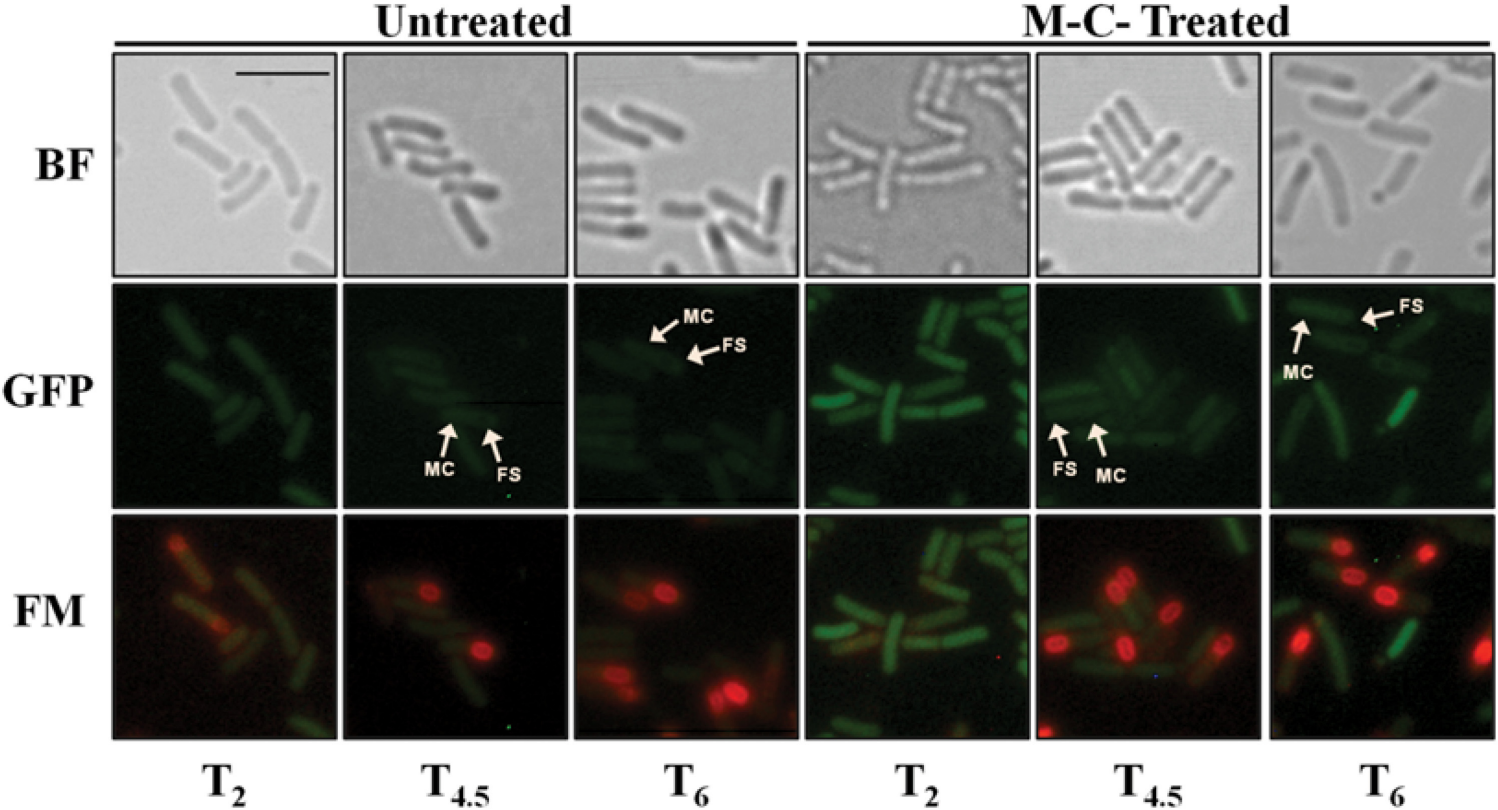
Monitoring of activation of the SOS response induced by DNA damage using a P_*recA*_-*gfp*mut3a during sporulation. Sporulating cells of *B*. *subtilis* PERM1238 (P_*recA*_-*gfp*mut3a) collected at different times after T_0_, in the sporulation program (T_2_, T_4.5_ and T_6_) were treated with M-C (125 ng/mL), incubated for additional 1 h and finally observed under fluorescence microscopy. BF, bright field; GFP, GFP channel; FM, FM4-64 staining; MC and FS, mother cell and forespore compartments. The scale bar is 2 μm and all images are at the same magnification.

To demonstrate that the SOS response is active during sporulation [11], the *B*. *subtilis* P_*recA*_*-gfpmut3a* strain was induced to sporulate, cell samples collected at 2, 4.5 and 6 hr after the onset of sporulation, were treated with M-C and then examined by fluorescence microscopy. The results of this analysis showed that the DNA damage promoted by M-C induced the synthesis of the GFPmut3a protein in the three-sporulation stages analyzed. Interestingly, in all cases, the GFP signal was detected in both the mother cell and the forespore compartments (Fig 1), indicating that DNA damage can induce the RecA-dependent SOS response in both compartments of the sporangium. In contrast, low fluorescence was detected in untreated sporangia of the P_*recA*_*-gfpmut3a* strain that were collected at the same developmental stages (Fig 1).

**Fig 2:**
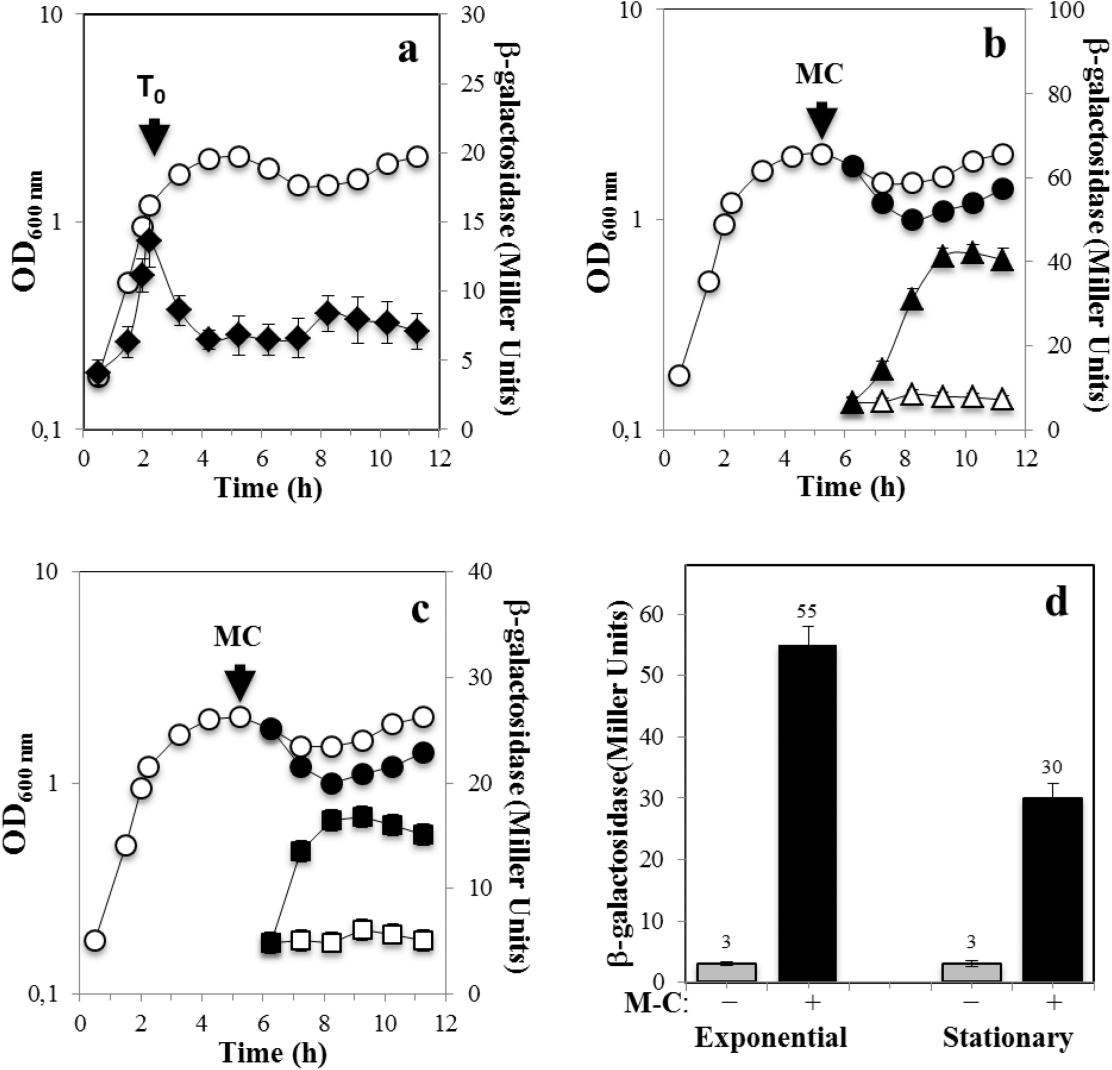
Levels of ß-galactosidase from *recA-lacZ* in growth and sporulation and with and without DNA damage. *B*. *subtilis* strain YB3001 containing a *recA-lacZ* fusion was grown and induced to sporulate in DSM (a-c) The optical densities of cultures were measured without (○) or after (●) DNA damaging treatment. Samples were also collected at different times during growth and sporulation and were processed and assayed for ß-galactosidase specific activity. In **(a)** ß-galactosidase from *recA-lacZ* was assayed throughout growth and sporulation (◆). In **(b,c)** 4 h after the onset of sporulation (T_0_), the culture was divided into two subcultures; one subculture was challenged with M-C (500 ng/mL) and the other one was untreated. Cells samples from untreated (open symbols) or treated (filled symbols) were collected at the indicated times and ß-galactosidase specific activity in the mother cell (b, triangles) and forespore (c, squares) fractions was determined, all as described in Materials and Methods. In (d), *B*. *subtilis* YB3001 was propagated in PAB medium, when the culture reached an OD_600nm_ = 0.5 (Exponential) or 4 h after T_0_ (Stationary), vegetative cells were treated (black bars) or not (gray bars) with M-C (500 ng/mL) for 1.5 h and then the cultures were processed for determination of ß-galactosidase as described above. Results are the average of values from three independent experiments ± standard deviations (SD) of ß-galactosidase specific activity.

To better assess and quantify the *recA* expression during the life cycle of *B*. *subtilis*, we used a strain carrying a transcriptional *recA-lacZ* fusion and determined the levels of ß-galactosidase produced during exponential growth and sporulation and in both the mother cell and forespore compartments. The results of this analysis revealed that the *recA*-driven ß-galactosidase activity was present in exponentially growing cells as well as in developing sporangia (Fig 2a). However, the expression levels of the *recA-lacZ* fusion were significantly lower in sporulation than during logarithmic growth (Fig 2a). These results are in agreement with a previous study that detected a lower amount of RecA in dormant spores than in vegetative cells [16]. Moreover, as shown in Fig 2d, addition of M-C to exponentially growing cells increased ~18 fold the expression levels of the *recA-lacZ* fusion.

**Fig 3:**
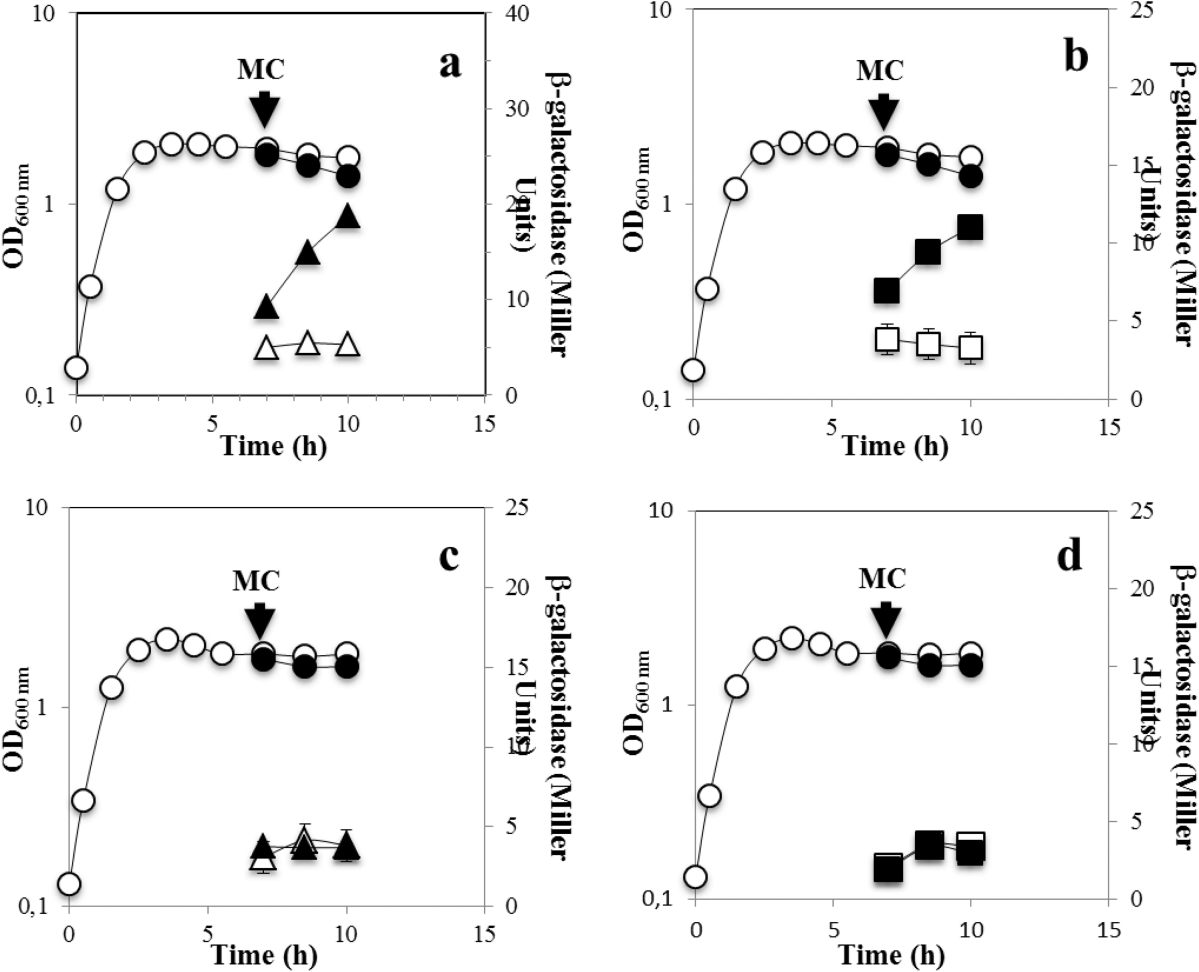
Levels of ß-galactosidase from *recA-lacZ* with and without DNA damage during sporulation in SOS-proficient (a and b) and –deficient (c and d) *B*. *subtilis* strains. *B*. *subtilis* strains LAS600 (parental) and LAS523 (SOS-deficient) containing a *recA-lacZ* fusion were grown and induced to sporulate in DSM. The optical densities of cultures were measured without (○) or after (●) DNA damaging treatment. 4 h after the onset of sporulation (T_0_), the culture was divided into two subcultures; one subculture was challenged with M-C (500 ng/mL) and the other one was untreated. Cells samples from untreated (open symbols) or treated (filled symbols) were collected at the indicated times and ß-galactosidase specific activity in the mother cell (a and c, triangles) and forespore (b and d, squares) fractions was determined, all as described in Materials and Methods.

A recent report showed that M-C was able to induce the expression of a chromosomal *uvrA-lacZ* fusion during stage IV of sporulation suggesting that the SOS response may be triggered in this stage of sporulation [11]. To further examine this suggestion, the *recA-lacZ* strain was treated with M-C 4 h after the onset of sporulation. Notably, in comparison with an untreated sporulating culture, addition of this alkylating agent increased the expression of the *recA-lacZ* 6- and 3-fold in the mother cell and the forespore compartment fractions, respectively (Fig 2b and 2c). Taken together, these results support the notion that in *B*. *subtilis* cells committed to sporulate, the SOS response can be activated and is possibly required to induce the expression of DNA repair proteins necessary to eliminate genetic insults that may compromise spore formation. In support of this contention, expression of the *recA-lacZ* fusion in both sporangia compartments was abolished in a mutant strain of *B*. *subtilis* unable to trigger the SOS response as it carries a non-cleavable form of the SOS-repressor protein DinR [31] (Fig 3).

Of note, in comparison with exponentially growing cells (Fig 2d), the extent of induction of the *recA-lacZ* fusion by M-C in strain YB3001 was ~2 fold lower during sporulation (Fig 2b and 2c) as well as during the stationary phase in vegetative cells propagated in PAB, a rich medium unable to promote *B*. *subtilis* sporulation [32] (Fig 2d). Therefore, the SOS-response can be elicited in *B*. *subtilis* despite unfavorable metabolic conditions prevailing in cells committed to sporulation or facing nutritional stress.

### Inactivation of *recA* delays sporulation

**Fig 4:**
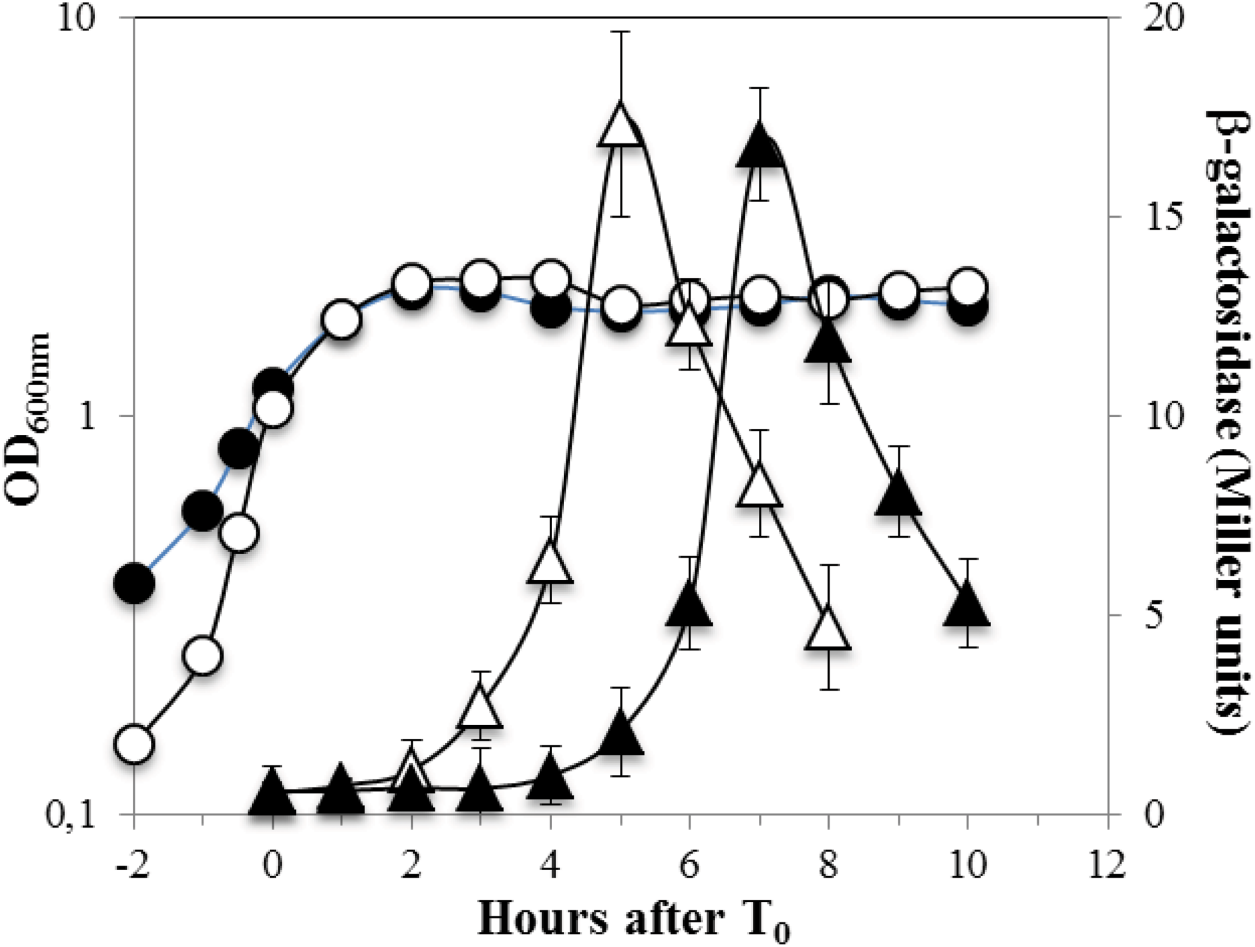
Expression of *spoVFA-lacZ* during sporulation of wild-type and Δ*recA B*. *subtilis* strains. *B*. *subtilis* strains PERM1233 (wild-type) (open symbols) and PERM1280 (**Δ***recA*) (closed symbols) containing the *spoVFA-lacZ* fusion were grown and induced to sporulate in DSM and the OD_600nm_ (circles) was measured. Cells were collected during sporulation at the indicated times, treated with lysozyme and the extracts were assayed for ß–galactosidase in the mother cell fractions only (triangles). Results are the averages of values from three independent experiments ± SD of ß-galactosidase specific activity.

It has been reported that a RecA-deficient *B*. *subtilis* strain exhibits morphological irregularities and a delayed prespore nucleoid condensation during sporulation [15], suggesting that RecA is required for normal sporulation. Therefore, we investigated further whether the lack of RecA interferes with growth and sporulation in *B*. *subtilis*. Our results showed that the growth rate of a Δ*recA* strain in DSM at 37°C had a significantly longer doubling time compared to the wild-type strain (33 ± 2 min and 24 ± 2 min, respectively; unpaired *t*-test, *P* < 0.05). Moreover, as previously described [15], the sporulation efficiency in the RecA-deficient strain was ~40% lower than that of the wild-type strain (data not shown). To directly examine effects of loss of RecA on sporulation-specific gene expression, we examined the expression of a *spoVFA-lacZ* fusion was used as a marker of gene expression late in sporulation of wild-type and Δ*recA* strains. The *spoVFA* gene encodes for subunit A of the dipicolinic acid (DPA) synthetase [33], and is expressed exclusively in the mother cell compartment reaching its highest expression level at T_5_ of sporulation [33, 34]. Analysis of *spoVFA*-*lacZ* expression during sporulation of the wild-type and Δ*recA* strains revealed a ~2 h delay in expression of *spoVFA*-*lacZ* fusion in the Δ*recA* mutant compared to the wild-type parental strain (Fig 4). Therefore, the morphological abnormalities observed in RecA-deficient sporangia [15] seem to be accompanied and presumably caused by alterations in the program of gene expression that drives the process of sporulation in *B*. *subtilis*. Notably, a recent report showed that *B*. *subtilis* cells expressing a non-phosphorylatable RecA protein also exhibited slowed sporulation [20]. Taken together, these results highlight the importance of RecA in this developmental process, as a deficiency in this protein slows sporulation significantly, presumably as a consequence of the resulting transcriptional abnormalities.

### RecA protects *B*. *subtilis* sporulating cells from DNA damage

In stage T_4.5_ where sporangial cells are commited to sporulate, the mother cell is programmed to lyse whereas forespores will eventually give rise to a dormant spore [2]. Therefore surviving colonies arising after treatment of T_4.5_ sporulating cells with genotoxic agents are mostly generated from forespores [10]. Keeping these facts in mind, to investigate whether RecA contributes to the survival of sporulating cells treated with DNA damaging agents, we examined the resistance of wild-type and Δ*recA B*. *subtilis* sporangia to M-C and UV-C radiation.

**Fig 5:**
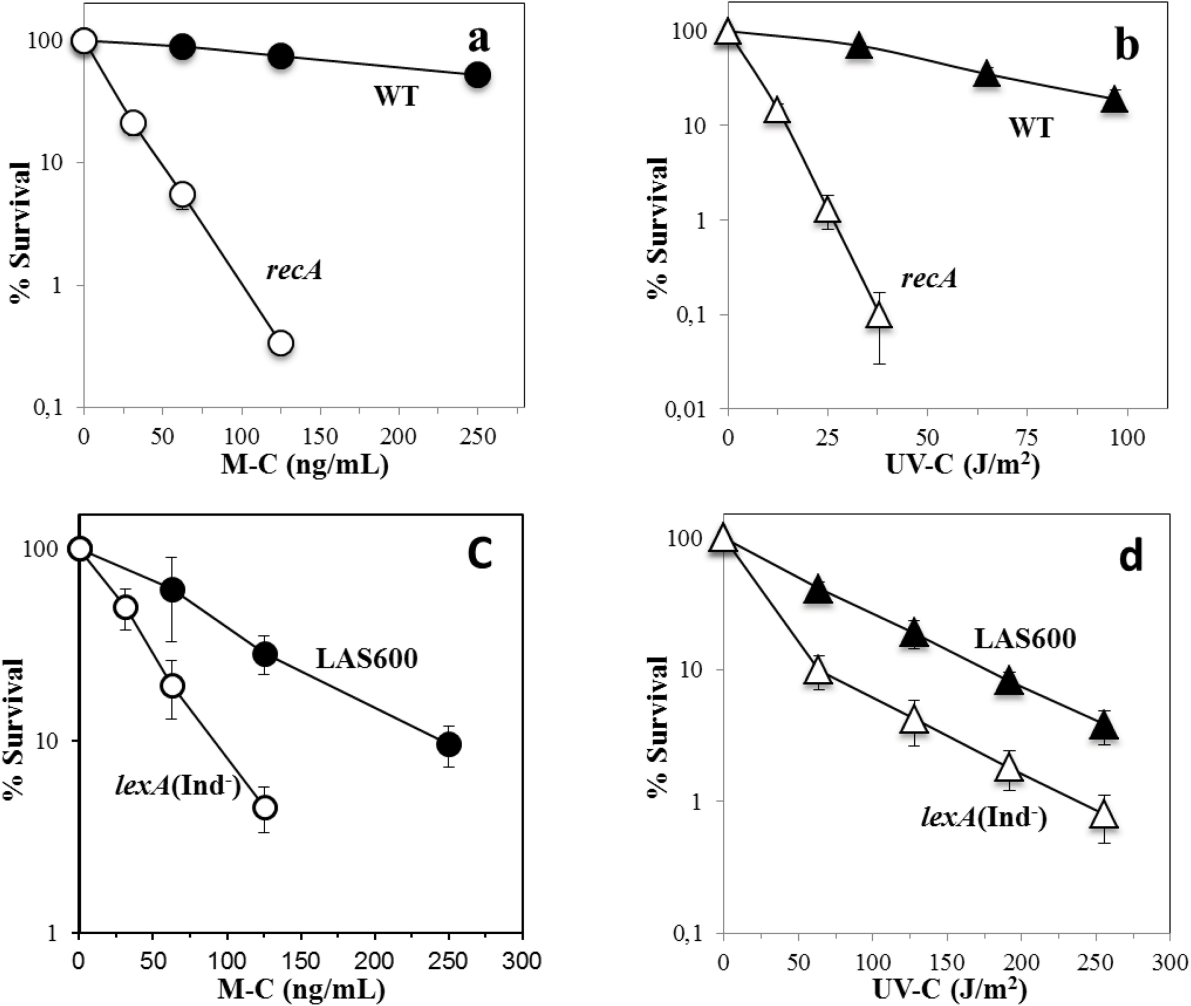
Resistance of Δ*recA* and SOS-deficient strains to M-C (a, c) and UV-C radiation (b, d) during sporulation. Sporulating cells of strains 168 (wild-type), PERM1030 (**Δ***recA*), LAS600 (parental) and LAS523 (*lexA*[ind^-^]) were treated (open symbols) or not (filled symbols) with increasing doses of M-C (a and c, circles) or UV-C light (b and d, triangles) at 4.5 h (wild-type, LAS600 and LAS523) or 6.5 h (Δ*recA*) after the onset of sporulation, and cell survival was determined as described in Materials and Methods. Data are expressed as the average ± SD of at least three independent experiments.

As noted above, the RecA-deficient strain is slowed ~2 h in sporulation. Therefore, in these experiments sporulating cells of the *recA* and parental strains were collected at equivalent morphological stages in sporulation (stages IV-V), as assessed by microscopic analysis. As shown in Fig 5a and 5b, in comparison with the wild-type strain the *recA* mutant exhibited an increased susceptibility to both M-C and UV-C radiation during sporulation. These two agents are known to produce lesions that block the progress of transcription elongation complexes [35, 36]. Notably, a previous report showed that the TCR factor Mfd plays a key role in protecting sporulating cells against M-C and UV-C [11], and the sporangia´s susceptibility to these genotoxic agents was greater in the Δ*recA* mutant than in the Mfd-deficient strain (Table 2). Together, these results strongly suggest that RecA as well as Mfd are necessary during spore morphogenesis to process pyrimidine dimers as well as DNA crosslinks generated by UV-C and M-C, respectively.

**Table 2:** Killing of *B*. *subtilis* forespores of various strains by DNA-damaging treatments.

Strain	LD_90_^a^	
	M-C (ng/mL)	UV-C (J/m^2^)
WT (168)	1,347 ± 74.4 ^*b*^	119.2 ± 14.6 ^*b*^
PERM938 (Δ*mfd*)	187.7 ± 13.2 ^*c*^	20.8 ± 3.9 ^*c*^
PERM1030 (Δ*recA*)	53.1 ± 5.7 ^*d*^	16.1 ± 3.7 ^*c*^
PERM1PP033 (Δ*mfd recA*)	12.7 ± 2.9 ^*e*^	3.9 ± 1.1 ^*d*^

^*a*^ *B*. *subtilis* sporangia from stage T_4.5_ were exposed to different concentrations of M-C or different doses of UV-C irradiation in order to determine the lethal dose to kill 90% of initial colony forming units (LD_90_). Results are expressed as averages ± SD of at least three independent experiments.

Superscripts *b*, *c*, *d* and *e* indicate statistically significant differences between strains under the same treatment as determined by one-way ANOVA followed by a Tukey’s *post hoc* test; *P* < 0.05.

Since cells committed to sporulate no longer replicate the chromosomes in the mother cell and the forespore compartments [2, 23], a recombination-repair role for RecA in this developmental stage can largely be ruled out. Rather, as shown in this work, the function of RecA is most likely to activate the SOS response to provide *B*. *subtilis* sporangia with the repair machinery to process DNA lesions occurring during sporulation. The experiments in Fig 5c and 5d provided further support for this contention by showing that in comparison with its parental strain (*B*. *subtilis* LAS600), sporulating cells of the SOS-deficient *lexA* (Ind^-^) mutant exhibited a major susceptibility to UV-C and M-C. Consistent with a previous report in dormant spores treated with ionizing radiation [19], our results revealed that sporangia harboring the *lexA* (Ind^-^) allele were less susceptible to M-C and UV-C than RecA-deficient sporangia (Fig 5). These analyses, also found that the SOS-deficient sporangia exhibited a higher resistance to UV-C than to M-C. However, in addition to the NER pathway sporulating cells rely on Mfd as well as an alternative excision repair pathway to counteract the noxious effects of UV-C [10, 11].

Since spore formation in *B*. *subtilis* requires the activation of hundreds of genes, it seems possible that most if not all DNA repair activity during this developmental process will be dedicated to eliminate damage from actively transcribing genes. Thus DNA repair proteins induced in the SOS response may work not only alone, but also in concert with Mfd as indicated by the following evidence. First, *uvrA*, a component of the NER machinery and a member of the SOS regulon, was induced by DNA damage in both sporangial compartments [11]. In addition, the absence of this essential NER component sensitized sporulating cells to UV-C and M-C and the inactivation of both Mfd and NER made sporangia even more sensitive to these agents [11]. Second, a UV-endonuclease encoded by *ywjD* operates through an alternative excision repair mechanism to eliminate UV-induced DNA damage in sporulating cells and dormant spores of *B*. *subtilis* [10]. Of note, a previous report have revealed that in addition to the spatio-temporal program that drives *ywjD* expression during sporulation [10], this gene is induced during spore germination by UV light in a RecA-independent manner [37]. Third, a recent study suggested that under conditions that overwhelm the repair capacity of the NER system, YwjD may repair UV-C-promoted lesions in an error-prone manner employing the polymerase activity of the low-fidelity enzymes YqjH and/or YqjW [11]. Indeed, YqjW also belongs to the SOS regulon [38], and is operative during *B*. *subtilis* sporulation and protects the forespore genome against mutagenic effects of UV-C irradiation [9].

Interestingly, as shown in Table 2, in comparison with sporangia lacking either RecA or Mfd, the absence of both functions increased the sporangia´s susceptibility to M-C and UV-C irradiation even more. These results strongly suggest that RecA and Mfd work independently to eliminate MC and UV-C promoted lesions; although it is also possible that by triggering the SOS response, RecA collaborates in the Mfd-mediated TCR pathway to eliminate genetic insults that may interfere with spore development.

Finally, as demonstrated in this work, RecA by triggering the SOS response provides the sporulating cells with the repair machinery necessary to eliminate genetic DNA damage. However, DNA lesions that are left unrepaired in the forespore compartment of sporangia or accumulated during dormancy are also eliminated during spore germination/outgrowth with participation of RecA in order to guarantee an ‘appropriate return’ to life of the dormant spores [16, 19, 39].
